# Atlas on substrate recognition subunits of CRL2 E3 ligases

**DOI:** 10.18632/oncotarget.8732

**Published:** 2016-04-14

**Authors:** Siwei Wang, Wenjia Xia, Mantang Qiu, Xin Wang, Feng Jiang, Rong Yin, Lin Xu

**Affiliations:** ^1^ Department of Thoracic Surgery, Nanjing Medical University Affiliated Cancer Hospital, Jiangsu Key Laboratory of Molecular and Translational Cancer Research, Cancer Institute of Jiangsu Province, Nanjing, China; ^2^ The Fourth Clinical College of Nanjing Medical University, Nanjing, China

**Keywords:** cullin-ring ligase(CRL), cullin2, E3 ligase, substrate recognition subunit (SRS)

## Abstract

The Cullin2-type ubiquitin ligases belong to the Cullin-Ring Ligase (CRL) family, which is a crucial determinant of proteasome-based degradation processes in eukaryotes. Because of the finding of von Hippel-Lindau tumor suppressor (VHL), the Cullin2-type ubiquitin ligases gain focusing in the research of many diseases, especially in tumors. These multisubunit enzymes are composed of the Ring finger protein, the Cullin2 scaffold protein, the Elongin B&C linker protein and the variant substrate recognition subunits (SRSs), among which the Cullin2 scaffold protein is the determining factor of the enzyme mechanism. Substrate recognition of Cullin2-type ubiquitin ligases depends on SRSs and results in the degradation of diseases associated substrates by intracellular signaling events. This review focuses on the diversity and the multifunctionality of SRSs in the Cullin2-type ubiquitin ligases, including VHL, LRR-1, FEM1b, PRAME and ZYG11. Recently, as more SRSs are being discovered and more aspects of substrate recognition have been illuminated, insight into the relationship between Cul2-dependent SRSs and substrates provides a new area for cancer research.

## INTRODUCTION

### Ubiquitin-proteasome system

**Figure 1:**
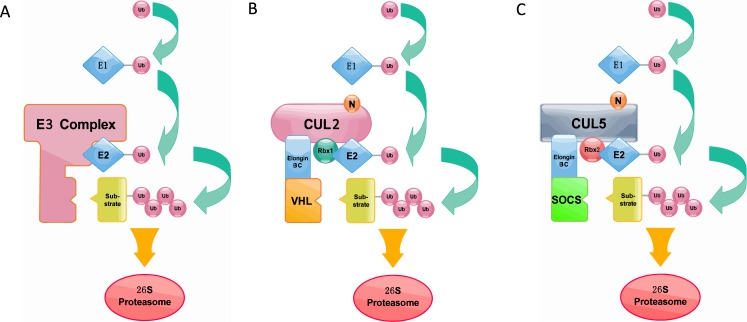
**A.**The process of ubiquitin transfer and ubiquitin-dependent degradation. **B.** Cullin2-type ubiquitin ligase E3 (ECV complex). Cul2 is used as a scaffold protein, and the Elongin BC complex connects the VHL box protein. **C.** Cullin5-type ubiquitin ligase E3 (ECS complex). Cul5 is used as a scaffold protein, and the Elongin BC complex connects the SOCS box protein. Like the ECV complex, the ECS complex also uses Ring box proteins to catalyze ubiquitin polymerization.

The ubiquitin-proteasome system is a crucial determinant of virtually all biological processes in eukaryotes and has emerged as a central mechanism to regulate protein turnover spatially and temporally [1, 2]. In this system, ubiquitin is covalently linked to a target protein through an enzymatic cascade, and the assembly of a poly-ubiquitin chain typically specifies the target protein for rapid degradation via 26S proteasome [3]. The process of ubiquitin transfer requires the activity of ubiquitin to orderly activate enzyme E1, ubiquitin-conjugating enzyme E2 and ubiquitin ligase E3 [4, 5]. Ubiquitin will be eventually linked to the substrate via an isopeptide bond between the C-terminal glycine of ubiquitin and a selected lysine residue of the substrate [6]. The repeated transfer of additional ubiquitin molecules to successive lysines on each previously conjugated ubiquitin generates a polyubiquitin chain [6, 7]. The polyubiquitin tag, a chain of at least four ubiquitin monomers, is recognized by the 26S proteasome where a host of protease sites rapidly degrade the protein into short peptides [8, 9] (Figure 1A). The specificity of ubiquitin-dependent proteolysis is derived from the many hundreds of E3 ubiquitin ligases that recognize a particular substrate through dedicated interaction domains [10]. Targeting motifs on substrates are typically short primary sequence elements that are often referred to as degrons [11]. Since the ubiquitin-proteasome system controls the stability of numerous regulators including cell cycle proteins, transcription factors, tumor suppressor proteins, oncoproteins, and membrane proteins [12–16], it therefore evoked lots of interests in the past decades.

### Cullin-ring ubiquitin ligases (CRLs)

As E3 enzymes are the core effectors of ubiquitin-proteasome system, it is reasonably important to fully dissect their molecular architecture. Currently we have known that Cullin scaffold subunits, containing at least five conserved Cullin family members, were the core subunits of E3 enzymes [17, 18]. All metazoans appear to have Cullins. They combine with RING finger proteins Rbx1/Roc1 to form highly diverse complexes called Cullin-RING ubiquitin ligases (CRLs) which play important roles in myriad cellular processes [19]. Members of CRLs function in a wide range of dynamic cellular processes, including the cell cycle, signal transduction, and transcription. And CRLs exhibit a conserved overall architecture that has plasticity to fine-tune the specific recruitment of different cullins [20].

Notably, Cullin2 is one of the best studied Cullin family members. It functions as a scaffolding protein and together with Ring protein constitutes the Cullin2-Ring ubiquitin ligase (CRL2), which plays important role in occurrence and progression of tumors [21].

### Substrate recognition subunits (SRS)

In addition to Cullins, substrate recognition subunits (SRS) are another core component of E3 ubiquitin ligase complexes. SRS is usually Cullin-dependent and exerts determinate effect on specific substrates degradation. Given the importance of Cullin2, there have been five confirmed Cul2-dependent SRSs discovered in succession during the past decades: the von Hippel-Lindau (VHL) tumor suppressor, which degrades hypoxia-inducible factor-α (HIF-α) under normoxic conditions [22]; LRR-1, which was first found to suppress 4-1-BB receptor signaling in CD4^+^ and CD8^+^ T cells [23], and then found to act as a SRS of Cullin2-type ubiquitin ligase [24]; FEM1b, which regulates glucose-stimulated insulin secretion [25]; PRAME, which is a transcription factor essential for early embryonic development that is confirmed to be enriched at enhancers and at transcriptionally active promoters [26]; and ZYG11, which was found to act as a cell-cycle regulator in Caenorhabditis elegans [27]. Recently, our group demonstrated that ZYG11A serves as an oncogene in non-small cell lung cancer via regulating CCNE1 expression [28].

Therefore, in this review we will focus on summarizing above all five confirmed Cul2-dependent SRSs and their substrates. Based on current acknowledgment, we also aim to make an atlas illuminating how SRSs of Cul2 complexes diversify the functions of this remarkable E3 enzyme family in diverse diseases [34].

## ATLAS ON CUL2-DEPENDENT SUBSTRATE RECOGNITION SUBUNITS

### VHL-box: a specific motif of Cul2-SRSs

**Figure 2:**
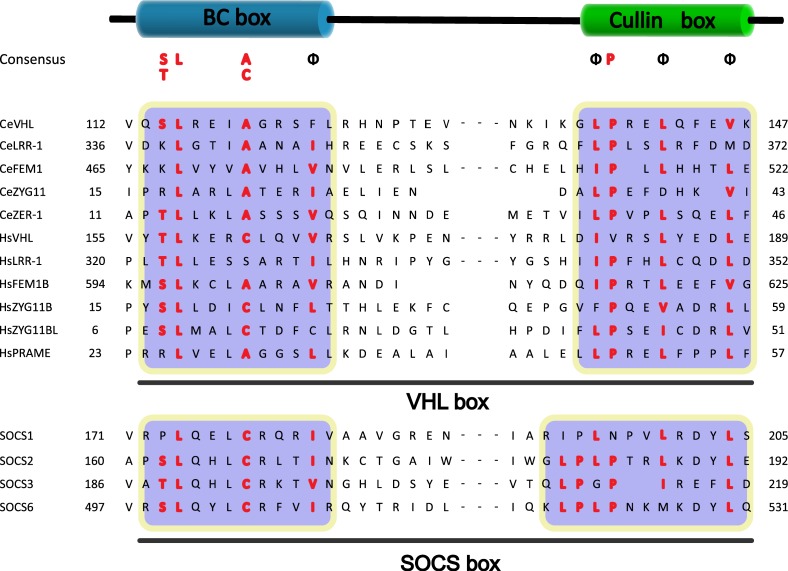
Sequence alignment of the VHL box and the SOCS box to Cul2 and Cul5 SRSs The regions for the BC box and Cullin box are marked. The conserved residues are highlighted, and the ΦPXXΦXXXΦ motif in the Cullin box is shown (Φ indicates a hydrophobic residue).

Before making the atlas of Cul2-dependent SRSs, a specific motif called VHL-box needs to be introduced at first. VHL-box was firstly identified in a well-known Cul2-SRS, VHL protein. Subsequent researches demonstrate that VHL-box was a specific motif engaged to bind with CRL2 by all known Cul2-dependent SRSs. Therefore VHL-box has been considered as a specific characteristic of Cul2-dependent SRSs currently (Figure 2) [29–31].

It is worth to mention that VHL was originally implicated as a SOCS-box which is considered as a specific motif of CRL5-SRSs. However, VHL lacks the C-terminal sequence (downstream of the BC box) of the SOCS box [24]. So, CRL2 associated SOCS-box proteins contain VHL-box in a strict sense. In order to differentiate CRL2 and CRL5-based SRSs, the general SOCS-boxcontaining proteins were further divided into two groups, CRL2 and CRL5 proteins [24]. The classical SOCS box-containing protein VHL-box is now classified as a Cul2-type protein, namely the VHL-Box protein. And the SOCS-box proteins are classified as the Cullin5-type protein [32]. Elongin B, Elongin C, SOCS-box and Cullin compose the complex (Figure 1C). Persons thus propose renaming the Elongin BC-Cul2-VHL-box protein and the Elongin BC-Cul5-SOCS-box protein complexes as the ECV and ECS complexes, respectively [33, 34]. Additionally, studies have provided direct biochemical evidence that the Cul2-box and the Cul5-box are parts of VHL-box and SOCS-box, respectively [33]. Together with ElonginBC-box, Cul2-box and Cul5-box make up the complete domains (Figure 2).

Generally speaking, VHL-box is specifically and functionally encoded in all known five Cul2-dependent SRSs described below. It is a feasible approach to identify novel Cul2-dependent SRSs by screening VHL-box.

### VHL

The VHL gene was identified in 1993 as the tumor suppressor gene whose germ line mutations were associated with the inherited von Hippel-Lindau cancer syndrome [35–37]. VHL mainly consists of two independent domains (domain α & β) that are involved in two independent functions: mediating nuclear export of the ECV complex and binding to substrate proteins [38]. Mutations of the VHL gene are associated with Von Hippel-Lindau disease, which is a hereditary cancer syndrome caused by germline mutations in the VHL tumor suppressor gene [36]. Most pathogenic VHL mutations inhibit formation of the ECV complex [39, 40]. More than 70% of VHL disease and sporadic clear cell renal carcinomas are caused by mutations or deletion of the BC box, which reduces binding affinity to the Elongin BC complex [41]. And then, the pVHL is found to function in the E3 ubiquitin ligase complex [42].

This complex is involved in the ubiquitination and degradation of the hypoxia-inducible factor (HIF), which is a transcription factor that plays a central role in the regulation of gene expression by oxygen [43]. HIF is necessary for tumor growth because most cancers have high metabolic demands and are supplied by structurally or functionally inadequate vasculature [44, 45]. Activation of HIF allows for enhanced angiogenesis, which in turn allows for increased glucose intake [46]. While HIF is mostly active in hypoxic conditions, VHL-defective renal carcinoma cells show constitutive activation of HIF even in oxygenated environments. It is clear that VHL and HIF interact closely [47]. Actually, the pVHL is so multifunctional that it could take on the functions of more types of substrates than HIF-α. Epidermal growth factor receptor (EGFR) is targeted by pVHL for polyubiquitination and degradation [48]. VHL could also suppress basal levels of Vascular Endothelial Growth Factor (VEGF) expression, restore hypoxia-inducibility of VEGF expression, and inhibit tumor formation in nude mice [49, 50]. Sprouty (Spry) proteins modulate the actions of receptor tyrosine kinases during development and tumorigenesis and are regulated by pVHL with ubiquitylation and degradation [51]. The 13 mammalian UBX-domain proteins in p97 are found to be linked to the VHL-dependent ubiquitin ligase E3 and its substrate hypoxia-inducible factor 1α (HIF1α) [52, 53]. Some other substrates such as Atypical PKC, RPB7 and Rpb1 could also be ubiquitylated and degraded by pVHL [47–50, 54]. The diversity in choosing substrates of CRL2^VHL^ makes Cullin2-type ubiquitin ligases multifunctional, and all above-mentioned downstream substrates of pVHL-Cul2 complexes are associated with diseases, especially in cancers. The complicated relationship between substrates still warrants much more research.

Coimmunoprecipitation and chromatographic copurification data suggest that pVHL-Cul2 complexes exist in native cells [42, 55]. And the crystal structure of the VHL protein reveals that the Elongin BC box of VHL binds to ElonginC [56]. Meanwhile, VHL was reported to bind, via Elongin C, to the human homolog of the C. elegans CUL2 protein. This ligation domain, which was originally called the “SOCS-box”, was then defined as the “VHL-box” [56]. The subsequently research indicates that a domain of the VHL protein was found to be bound to Elongin B and Elongin C, and this component was defined as the “VHL box”, which is composed of an Elongin BC box and a Cul2 box [24, 57]. Further study demonstrated that the Cul2 box is located C-terminal to the Elongin BC box and contains the consensus sequence ΦPXXΦXXXΦ, where the first position is most frequently a leucine [33] (Figure 2). The subsequent researches in mammalian revealed that SRSs (e.g., VHL, FEM1b, and LRR-1) bound to Elongin BC are all combined with a three α-helix structure, which is actually a component of the VHL box [58]. According to the amino acid sequence analysis, the VHL-boxes in different proteins are similar and highly conserved (Figure 2).

### LRR1

Leucine-rich repeat protein 1 (LRR-1) is an essential determinant of genome stability in C. elegans that acts as a substrate recognition subunit of a CRL2 complex (CRL2^LRR-1^). LRR-1 is a nuclear protein that contains a typical Elongin BC and Cul2 box, which is the signature of Cullin2-type ubiquitin ligase SRSs; LRR-1 binds through this motif both *in vitro* and *in vivo* [59].

The accumulation of CKI-1 in C. elegans was found to be correlated with Cul2 mutant germ cells, which undergo a G1-phase arrest [60]. And then it was discovered that nematode LRR-1 degrades the Cip/Kip CDK-inhibitor (CKI) p21^Cip1^ in C. elegans to ensure G1-phase cell cycle progression in germ cells [61]. Human LRR-1 also polyubiquitinates and degrades the CKI p21^Cip1^ but it does not affect cell cycle progression [61]. In contrast, human Cul2^LRR1^ acts as a critical regulator of cell motility that promotes a nonmotile stationary cell state by preventing p21 from inhibiting the Rho/ROCK/LIMK pathway [61]. These data indicate that human LRR-1 is a negative regulator of cofilin, a protein that decreases cell motility [61].

The later research also indicates that LRR-1 acts as a nuclear substrate-recognition subunit of a CRL2 complex, which ensures DNA replication integrity [59]. Loss of LRR-1 function induces re-replication of DNA and causes the accumulation of stretches of ssDNA, which leads to cell cycle arrest in the mitotic region of the germ line. SsDNA-RPA-1 nuclear foci then recruit and activate ATL-1, which, together with the CHK-1 kinase, prevents CDK-1 activation (dephosphorylation via CDC-25) and cell cycle progression [59, 62]. Collectively, LRR-1 inactivation leads to activation of the ATL-1/CHK-1 (the C. elegans orthologues of ATR/Chk1) pathway, which delays mitotic entry and results in embryonic lethality [59, 63]. CRL2^LRR-1^ also participates in the mitotic proliferation/meiotic entry decision and inhibits the first steps of meiotic prophase by targeting in mitotic germ cells the degradation of the HORMA domain-containing protein HTP-3, which is required for loading synaptonemal complex components onto meiotic chromosomes. [64]

In conclusion, as the most recently identified SRSs of the CRL2 complex family, few downstream products and pathways have been confirmed and more work is needed to determine additional details of the CRL2^LRR-1^-mediated ubiquitin proteolysis. Same as pVHL, studies of CRL2^LRR-1^ in diseases warrants much more research. According to the results in germ cell lines, hopes are high for the outcome of the joint research of CRL2^LRR-1^ in diverse tumors.

### FEM1

The mammalian Fem1b gene encodes a homolog of FEM-1, a protein in the sex-determination pathway of nematode Caenorhabditis elegans. The pathway controlling sex determination in the nematode is a model for the genetic control of cell-fate determination [65]. Fem1b and FEM-proteins each contain a VHL-box motif that mediates their interaction with certain E3 ubiquitin ligase complexes [66, 67]. A study also indicated that there may be evolutionary conservation of the regulation and function between the mouse and human FEM1B genes [68].

In C. elegans, FEM-1 negatively regulates the Gli-family transcription factor TRA-1, which is the terminal effector of the sex-determination pathway, and functions as a CRL2 complex SRS to target TRA-1 for ubiquitylation [66, 69]. CRL2^FEM-1^ controls TRA-1-repressor activity through the degradation of full-length TRA-1A, and FEM-2 as well as FEM-3 increase the efficiency of FEM-1 mediated degradation of TRA-1A [69]. Ankyrin repeat domain 37 (Ankrd37), a protein containing ankyrin repeats and a putative nuclear localization signal, is reported to be targeted by FEM1b and degraded by FEM1b in the same manner as TRA-1 [70]. Overexpression of FEM-1 is found induces apoptosis in mammalian cells [71]. Additionally, the protein Fem1b is found to be downregulated by the proteasome in malignant colon cancer cells, and Fem1b increases proteasome inhibitor-induced apoptosis of these cells [72]. According to this research, FEM1b could represent a novel molecular target to overcome resistance to apoptosis in colon cancer [72].

### PRAME

We have introduced LRR-1 and FEM1B as Cul2-Rbx-interacting proteins that contain a VHL box as well as protein-protein interaction motifs (leucine-rich repeats and ankyrin repeats, respectively). Similar to LRR-1 and FEM1, preferentially expressed antigen of melanoma (PRAME) contains a VHL box. Researchers used protein-complex purification strategies and identified PRAME as a substrate recognition subunit of a Cullin2-based E3 ubiquitin ligase to confirm its physiological interaction with the endogenous Cul2-Rbx1 complex [24, 26].

Genome-wide chromatin immunoprecipitation experiments revealed that PRAME is specifically enriched at enhancers and at transcriptionally active promoters that are also bound by nuclear transcription factor Y (NFY), a transcription factor essential for early embryonic development and cell proliferation [26, 73]. Recently, a study mined the PRAME interactome to a deeper level and identified specific interactions with ATPase Kae1p (OSGEP) and LAGE3, which are human orthologues of the ancient EKC/KEOPS complex [74–77]. Moreover, EKC subunits associate with PRAME target sites on chromatin. The data reveal a novel link between the oncoprotein PRAME and the conserved EKC complex and support a role for both complexes in the same pathways [76, 78].

At present, overexpression of PRAME is frequently found in a wide variety of human cancers, including hematological tumors, lung, breast and renal carcinoma [79–81]. Although these findings suggested a role for PRAME in human malignancies, the detailed molecular mechanisms and pathways involved are not yet clear. From the perspective of CRL2^PRAME^-mediated ubiquitylation, a breakthrough is not far off.

### ZYG11

C. elegans has two ZYG11 family members, ZYG-11 and ZER-1; Drosophila has a single ZYG11 homologue; sea urchins have two ZYG11 homologues; and mice and humans have three ZYG11 homologues each (ZYG11A, ZYG11B and ZYG11BL). However, in mammals, ZYG11A and ZYG11B seem to have arisen by a recent duplication and are tandemly adjacent to each other in the mouse and human genomes. Using *in situ* hybridization and immunohistochemistry, the presence of ZYG11 expression was clearly established around the time of meiosis. The cell-specific expression of ZYG11 transcripts and the conservation of this gene among distant species suggest that this protein may play an important role during meiosis [82]. All of the ZYG11 family members contain an ARM-like helical domain and at least three vLRR (variant leucine-rich repeat) motifs [27, 82].

In C. elegans, ZYG11 was identified as a cell-cycle regulator. Studies establish that ZYG11 and CUL-2 promote the metaphase-to-anaphase transition and M phase arrest at meiosis II [27, 83, 84]. Anterior-posterior polarity (AP polarity) and protease-activated receptor 2 (PAR-2) are all bound up with ZYG-11 [85]. Research results of previous research indicate that ZYG-11 acts with a Cul-2-based E3 ligase that is essential during meiosis II and functions redundantly with the Anaphase-promoting Complex/Cyclosome (APC) in meiosis I. The data also indicate that delayed M phase results from the accumulation of the B-type cyclin CYB-3, which is regulated by APC, and CYB-3 is a target of the E3 ligase [83]. Meanwhile, the gene Mei-1 was discovered to possibly correspond to ZYG11, and Mei-1 promotes a non-disjunction of the chromosome at meiosis I [82]. In humans, ZYG11BL has high levels of expression in skeletal muscle and testes, in which it is expressed in late pachytene spermatocytes and spermatids [82].

ZYG11 has been shown to bind to the CUL-2 complex through direct interaction with Elongin C, and ZYG11 binds to Elongin C using a nematode variant of the VHL-box motif, which means that ZYG11 acts as the SRS for a CRL^ZYG11^ complex [27, 84]. Given the many essential functions carried out by ZYG-11 in C. elegans, ZYG11 homologues in humans are predicted to function in important CUL2-dependent cellular processes. The latest research indicates that ZYG11A may serve as a novel oncogene promoting tumorigenicity of NSCLC cells by inducing cell cycle alterations and increasing CCNE1 expression [28].

## DISCUSSION AND CLINICAL SIGNIFICANCE

**Figure 3:**
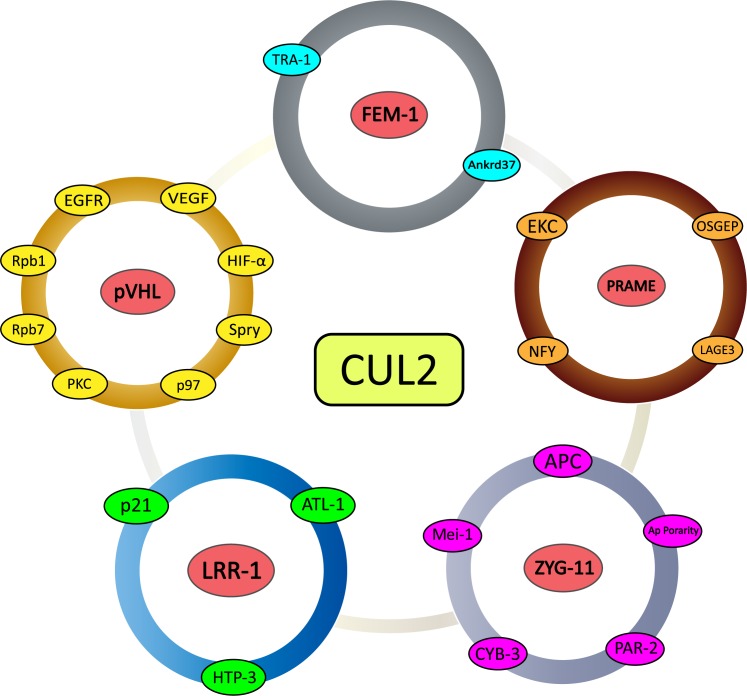
Schematic representation of CRL2-mediated substrate recognition subunits and their substrates All five of the examined substrate recognition subunits with detailed research dates (pVHL, FEM-1, PRAME, LRR-1, and ZYG11) and associated downstream direct and indirect substrates.

During the past few years, considerable progress has been made on the characterization of Cullin-based ligases. At the same time, remarkable strides have been made in the discovery of global cycles that are regulated and activated by Cullin2-based ligases in diseases, especially in tumors. In summary, these exciting discoveries highlight the extraordinary possibility of Cullin2-based ligases to target a very large number of substrates for ubiquitin-dependent degradation (Figure 3). Although many SRSs have been identified, further work is still needed to elucidate the relationship between SRSs and the myriad substrates in the intracellular signal transduction pathway. Similar to the inactivation of pVHL leading to the accumulation of HIF-α in renal carcinoma, the CRL2-mediated degradation pathways of the remaining SRSs and their substrates are also functioning in other diseases. Among them, the polyubiquitination and degradation of VEGF inhibits the formation of tumors. Some others such as P21 and EGFR, which have been studied thoroughly, are associated with a wide variety of diseases. Because pVHL is inactivated in von Hippel-Lindau cancer syndrome, researchers have conducted careful and thorough studies of the functions of Cullin2-based E3 ligases. As with pVHL, the remaining SRSs and substrates are mutated in some human diseases, and investigating their mechanisms with regard to a possible role in ubiquitin-dependent degradation pathways may be rewarding.

## References

[1] E AH, Heinz WF, Antonik MD, D'Costa NP, Nageswaran S, Schoenenberger CA, Hoh JH (1998). Relative microelastic mapping of living cells by atomic force microscopy. Biophys J.

[2] Shabek N, Ciechanover A (2010). Degradation of ubiquitin: the fate of the cellular reaper. Cell cycle.

[3] Hershko A, Heller H, Elias S, Ciechanover A (1983). Components of ubiquitin-protein ligase system. Resolution, affinity purification, and role in protein breakdown. The Journal of biological chemistry.

[4] Etlinger JD, Goldberg AL (1977). A soluble ATP-dependent proteolytic system responsible for the degradation of abnormal proteins in reticulocytes. Proceedings of the National Academy of Sciences of the United States of America.

[5] Lam YA, Lawson TG, Velayutham M, Zweier JL, Pickart CM (2002). A proteasomal ATPase subunit recognizes the polyubiquitin degradation signal. Nature.

[6] Pickart CM (2000). Ubiquitin in chains. Trends in biochemical sciences.

[7] Nandi D, Tahiliani P, Kumar A, Chandu D (2006). The ubiquitin-proteasome system. J Biosci.

[8] Baumeister W, Walz J, Zuhl F, Seemuller E (1998). The proteasome: paradigm of a self-compartmentalizing protease. Cell.

[9] Murata S, Yashiroda H, Tanaka K (2009). Molecular mechanisms of proteasome assembly. Nat Rev Mol Cell Biol.

[10] Eletr ZM, Wilkinson KD (2014). Regulation of proteolysis by human deubiquitinating enzymes. Biochimica et biophysica acta.

[11] Varshavsky A (1991). Naming a targeting signal. Cell.

[12] Peters JM, Franke WW, Kleinschmidt JA (1994). Distinct 19 S and 20 S subcomplexes of the 26 S proteasome and their distribution in the nucleus and the cytoplasm. The Journal of biological chemistry.

[13] Orlowski RZ (1999). The role of the ubiquitin-proteasome pathway in apoptosis. Cell death and differentiation.

[14] Petroski MD, Deshaies RJ (2005). Function and regulation of cullin-RING ubiquitin ligases. Nat Rev Mol Cell Biol.

[15] Sasagawa Y, Kikuchi K, Dazai K, Higashitani A (2005). Caenorhabditis elegans Elongin BC complex is essential for cell proliferation and chromosome condensation and segregation during mitosis and meiotic division II. Chromosome research.

[16] Hwang W, Artan M, Seo M, Lee D, Nam HG, Lee SV (2015). Inhibition of elongin C promotes longevity and protein homeostasis via HIF-1 in C. elegans. Aging cell.

[17] Deshaies RJ, Joazeiro CA (2009). RING domain E3 ubiquitin ligases. Annual review of biochemistry.

[18] Sarikas A, Hartmann T, Pan ZQ (2011). The cullin protein family. Genome Biol.

[19] Bosu DR, Kipreos ET (2008). Cullin-RING ubiquitin ligases: global regulation and activation cycles. Cell Div.

[20] Nguyen HC, Yang H, Fribourgh JL, Wolfe LS, Xiong Y (2015). Insights into Cullin-RING E3 ubiquitin ligase recruitment: structure of the VHL-EloBC-Cul2 complex. Structure.

[21] Kong X, Lin Z, Liang D, Fath D, Sang N, Caro J (2006). Histone deacetylase inhibitors induce VHL and ubiquitin-independent proteasomal degradation of hypoxia-inducible factor 1alpha. Molecular and cellular biology.

[22] Kim WY, Kaelin WG (2004). Role of VHL gene mutation in human cancer. Journal of clinical oncology.

[23] Jang LK, Lee ZH, Kim HH, Hill JM, Kim JD, Kwon BS (2001). A novel leucine-rich repeat protein (LRR-1): potential involvement in 4-1BB-mediated signal transduction. Mol Cells.

[24] Kamura T, Maenaka K, Kotoshiba S, Matsumoto M, Kohda D, Conaway RC, Conaway JW, Nakayama KI (2004). VHL-box and SOCS-box domains determine binding specificity for Cul2-Rbx1 and Cul5-Rbx2 modules of ubiquitin ligases. Genes & development.

[25] Lu D, Ventura-Holman T, Li J, McMurray RW, Subauste JS, Maher JF (2005). Abnormal glucose homeostasis and pancreatic islet function in mice with inactivation of the Fem1b gene. Molecular and cellular biology.

[26] Costessi A, Mahrour N, Tijchon E, Stunnenberg R, Stoel MA, Jansen PW, Sela D, Martin-Brown S, Washburn MP, Florens L, Conaway JW, Conaway RC, Stunnenberg HG (2011). The tumour antigen PRAME is a subunit of a Cul2 ubiquitin ligase and associates with active NFY promoters. The EMBO journal.

[27] Vasudevan S, Starostina NG, Kipreos ET (2007). The Caenorhabditis elegans cell-cycle regulator ZYG-11 defines a conserved family of CUL-2 complex components. EMBO reports.

[28] Wang X, Sun Q, Chen C, Yin R, Huang X, Wang X, Shi R, Xu L, Ren B (2016). ZYG11A serves as an oncogene in non-small cell lung cancer and influences CCNE1 expression. Oncotarget.

[29] Kipreos ET, Lander LE, Wing JP, He WW, Hedgecock EM (1996). cul-1 is required for cell cycle exit in C. elegans and identifies a novel gene family. Cell.

[30] Zheng N, Schulman BA, Song L, Miller JJ, Jeffrey PD, Wang P, Chu C, Koepp DM, Elledge SJ, Pagano M, Conaway RC, Conaway JW, Harper JW, Pavletich NP (2002). Structure of the Cul1-Rbx1-Skp1-F boxSkp2 SCF ubiquitin ligase complex. Nature.

[31] Goldenberg SJ, Cascio TC, Shumway SD, Garbutt KC, Liu J, Xiong Y, Zheng N (2004). Structure of the Cand1-Cul1-Roc1 complex reveals regulatory mechanisms for the assembly of the multisubunit cullin-dependent ubiquitin ligases. Cell.

[32] Kamura T, Sato S, Haque D, Liu L, Kaelin WG, Conaway RC, Conaway JW (1998). The Elongin BC complex interacts with the conserved SOCS-box motif present in members of the SOCS, ras, WD-40 repeat, and ankyrin repeat families. Genes & development.

[33] Mahrour N, Redwine WB, Florens L, Swanson SK, Martin-Brown S, Bradford WD, Staehling-Hampton K, Washburn MP, Conaway RC, Conaway JW (2008). Characterization of Cullin-box sequences that direct recruitment of Cul2-Rbx1 and Cul5-Rbx2 modules to Elongin BC-based ubiquitin ligases. The Journal of biological chemistry.

[34] Sasagawa Y, Sato S, Ogura T, Higashitani A (2007). C. elegans RBX-2-CUL-5- and RBX-1-CUL-2-based complexes are redundant for oogenesis and activation of the MAP kinase MPK-1. FEBS letters.

[35] Gnarra JR, Duan DR, Weng Y, Humphrey JS, Chen DY, Lee S, Pause A, Dudley CF, Latif F, Kuzmin I, Schmidt L, Duh FM, Stackhouse T, Chen F, Kishida T, Wei MH (1996). Molecular cloning of the von Hippel-Lindau tumor suppressor gene and its role in renal carcinoma. Biochimica et biophysica acta.

[36] Latif F, Tory K, Gnarra J, Yao M, Duh FM, Orcutt ML, Stackhouse T, Kuzmin I, Modi W, Geil L (1993). Identification of the von Hippel-Lindau disease tumor suppressor gene. Science.

[37] Ohh M, Kaelin WG (2003). VHL and kidney cancer. Methods in molecular biology.

[38] Bonicalzi ME, Groulx I, de Paulsen N, Lee S (2001). Role of exon 2-encoded beta -domain of the von Hippel-Lindau tumor suppressor protein. The Journal of biological chemistry.

[39] Clifford SC, Walsh S, Hewson K, Green EK, Brinke A, Green PM, Gianelli F, Eng C, Maher ER (1999). Genomic organization and chromosomal localization of the human CUL2 gene and the role of von Hippel-Lindau tumor suppressor-binding protein (CUL2 and VBP1) mutation and loss in renal-cell carcinoma development. Genes, chromosomes & cancer.

[40] Schoenfeld AR, Davidowitz EJ, Burk RD (2000). Elongin BC complex prevents degradation of von Hippel-Lindau tumor suppressor gene products. Proceedings of the National Academy of Sciences of the United States of America.

[41] Kishida T, Stackhouse TM, Chen F, Lerman MI, Zbar B (1995). Cellular proteins that bind the von Hippel-Lindau disease gene product: mapping of binding domains and the effect of missense mutations. Cancer research.

[42] Pause A, Lee S, Worrell RA, Chen DY, Burgess WH, Linehan WM, Klausner RD (1997). The von Hippel-Lindau tumor-suppressor gene product forms a stable complex with human CUL-2, a member of the Cdc53 family of proteins. Proceedings of the National Academy of Sciences of the United States of America.

[43] Maxwell PH, Wiesener MS, Chang GW, Clifford SC, Vaux EC, Cockman ME, Wykoff CC, Pugh CW, Maher ER, Ratcliffe PJ (1999). The tumour suppressor protein VHL targets hypoxia-inducible factors for oxygen-dependent proteolysis. Nature.

[44] Park SW, Chung NG, Hur SY, Kim HS, Yoo NJ, Lee SH (2009). Mutational analysis of hypoxia-related genes HIF1alpha and CUL2 in common human cancers. APMIS.

[45] Poon E, Harris AL, Ashcroft M (2009). Targeting the hypoxia-inducible factor (HIF) pathway in cancer. Expert reviews in molecular medicine.

[46] Zimna A, Kurpisz M (2015). Hypoxia-Inducible Factor-1 in Physiological and Pathophysiological Angiogenesis: Applications and Therapies. BioMed research international.

[47] Jaakkola P, Mole DR, Tian YM, Wilson MI, Gielbert J, Gaskell SJ, von Kriegsheim A, Hebestreit HF, Mukherji M, Schofield CJ, Maxwell PH, Pugh CW, Ratcliffe PJ (2001). Targeting of HIF-alpha to the von Hippel-Lindau ubiquitylation complex by O2-regulated prolyl hydroxylation. Science.

[48] Zhou L, Yang H (2011). The von Hippel-Lindau tumor suppressor protein promotes c-Cbl-independent poly-ubiquitylation and degradation of the activated EGFR. PloS one.

[49] Blankenship C, Naglich JG, Whaley JM, Seizinger B, Kley N (1999). Alternate choice of initiation codon produces a biologically active product of the von Hippel Lindau gene with tumor suppressor activity. Oncogene.

[50] Maeda Y, Suzuki T, Pan X, Chen G, Pan S, Bartman T, Whitsett JA (2008). CUL2 is required for the activity of hypoxia-inducible factor and vasculogenesis. The Journal of biological chemistry.

[51] Anderson K, Nordquist KA, Gao X, Hicks KC, Zhai B, Gygi SP, Patel TB (2011). Regulation of cellular levels of Sprouty2 protein by prolyl hydroxylase domain and von Hippel-Lindau proteins. The Journal of biological chemistry.

[52] Alexandru G, Graumann J, Smith GT, Kolawa NJ, Fang R, Deshaies RJ (2008). UBXD7 binds multiple ubiquitin ligases and implicates p97 in HIF1alpha turnover. Cell.

[53] den Besten W, Verma R, Kleiger G, Oania RS, Deshaies RJ (2012). NEDD8 links cullin-RING ubiquitin ligase function to the p97 pathway. Nature structural & molecular biology.

[54] Okuda H, Saitoh K, Hirai S, Iwai K, Takaki Y, Baba M, Minato N, Ohno S, Shuin T (2001). The von Hippel-Lindau tumor suppressor protein mediates ubiquitination of activated atypical protein kinase C. The Journal of biological chemistry.

[55] Lonergan KM, Iliopoulos O, Ohh M, Kamura T, Conaway RC, Conaway JW, Kaelin WG (1998). Regulation of hypoxia-inducible mRNAs by the von Hippel-Lindau tumor suppressor protein requires binding to complexes containing elongins B/C and Cul2. Molecular and cellular biology.

[56] Stebbins CE, Kaelin WG, Pavletich NP (1999). Structure of the VHL-ElonginC-ElonginB complex: implications for VHL tumor suppressor function. Science.

[57] Maher ER, Kaelin WG (1997). von Hippel-Lindau disease. Medicine.

[58] Gossage L, Pires DE, Olivera-Nappa A, Asenjo J, Bycroft M, Blundell TL, Eisen T (2014). An integrated computational approach can classify VHL missense mutations according to risk of clear cell renal carcinoma. Human molecular genetics.

[59] Merlet J, Burger J, Tavernier N, Richaudeau B, Gomes JE, Pintard L (2010). The CRL2LRR-1 ubiquitin ligase regulates cell cycle progression during C. elegans development. Development.

[60] Feng H, Zhong W, Punkosdy G, Gu S, Zhou L, Seabolt EK, Kipreos ET (1999). CUL-2 is required for the G1-to-S-phase transition and mitotic chromosome condensation in Caenorhabditis elegans. Nature cell biology.

[61] Starostina NG, Simpliciano JM, McGuirk MA, Kipreos ET (2010). CRL2(LRR-1) targets a CDK inhibitor for cell cycle control in C. elegans and actin-based motility regulation in human cells. Developmental cell.

[62] Encalada SE, Martin PR, Phillips JB, Lyczak R, Hamill DR, Swan KA, Bowerman B (2000). DNA replication defects delay cell division and disrupt cell polarity in early Caenorhabditis elegans embryos. Developmental biology.

[63] Brauchle M, Baumer K, Gonczy P (2003). Differential activation of the DNA replication checkpoint contributes to asynchrony of cell division in C. elegans embryos. Current biology.

[64] Burger J, Merlet J, Tavernier N, Richaudeau B, Arnold A, Ciosk R, Bowerman B, Pintard L (2013). CRL2(LRR-1) E3-ligase regulates proliferation and progression through meiosis in the Caenorhabditis elegans germline. PLoS genetics.

[65] Ventura-Holman T, Seldin MF, Li W, Maher JF (1998). The murine fem1 gene family: homologs of the Caenorhabditis elegans sex-determination protein FEM-1. Genomics.

[66] Gilder AS, Chen YB, Jackson RJ, Jiang J, Maher JF (2013). Fem1b promotes ubiquitylation and suppresses transcriptional activity of Gli1. Biochemical and biophysical research communications.

[67] Hodgkin J, Doniach T, Shen M (1985). The sex determination pathway in the nematode Caenorhabditis elegans: variations on a theme. Cold Spring Harb Symp Quant Biol.

[68] Ventura-Holman T, Maher JF (2000). Sequence, organization, and expression of the human FEM1B gene. Biochemical and biophysical research communications.

[69] Starostina NG, Lim JM, Schvarzstein M, Wells L, Spence AM, Kipreos ET (2007). A CUL-2 ubiquitin ligase containing three FEM proteins degrades TRA-1 to regulate C. elegans sex determination. Developmental cell.

[70] Shi YQ, Liao SY, Zhuang XJ, Han CS (2011). Mouse Fem1b interacts with and induces ubiquitin-mediated degradation of Ankrd37. Gene.

[71] Chan SL, Yee KS, Tan KM, Yu VC (2000). The Caenorhabditis elegans sex determination protein FEM-1 is a CED-3 substrate that associates with CED-4 and mediates apoptosis in mammalian cells. The Journal of biological chemistry.

[72] Subauste MC, Sansom OJ, Porecha N, Raich N, Du L, Maher JF (2010). Fem1b, a proapoptotic protein, mediates proteasome inhibitor-induced apoptosis of human colon cancer cells. Mol Carcinog.

[73] Bhattacharya A, Deng JM, Zhang Z, Behringer R, de Crombrugghe B, Maity SN (2003). The B subunit of the CCAAT box binding transcription factor complex (CBF/NF-Y) is essential for early mouse development and cell proliferation. Cancer research.

[74] Alpen B, Gure AO, Scanlan MJ, Old LJ, Chen YT (2002). A new member of the NY-ESO-1 gene family is ubiquitously expressed in somatic tissues and evolutionarily conserved. Gene.

[75] Downey M, Houlsworth R, Maringele L, Rollie A, Brehme M, Galicia S, Guillard S, Partington M, Zubko MK, Krogan NJ, Emili A, Greenblatt JF, Harrington L, Lydall D, Durocher D (2006). A genome-wide screen identifies the evolutionarily conserved KEOPS complex as a telomere regulator. Cell.

[76] Costessi A, Mahrour N, Sharma V, Stunnenberg R, Stoel MA, Tijchon E, Conaway JW, Conaway RC, Stunnenberg HG (2012). The human EKC/KEOPS complex is recruited to Cullin2 ubiquitin ligases by the human tumour antigen PRAME. PloS one.

[77] Kisseleva-Romanova E, Lopreiato R, Baudin-Baillieu A, Rousselle JC, Ilan L, Hofmann K, Namane A, Mann C, Libri D (2006). Yeast homolog of a cancer-testis antigen defines a new transcription complex. The EMBO journal.

[78] Srinivasan M, Mehta P, Yu Y, Prugar E, Koonin EV, Karzai AW, Sternglanz R (2011). The highly conserved KEOPS/EKC complex is essential for a universal tRNA modification, t6A. The EMBO journal.

[79] Ikeda H, Lethe B, Lehmann F, van Baren N, Baurain JF, de Smet C, Chambost H, Vitale M, Moretta A, Boon T, Coulie PG (1997). Characterization of an antigen that is recognized on a melanoma showing partial HLA loss by CTL expressing an NK inhibitory receptor. Immunity.

[80] Kilpinen S, Autio R, Ojala K, Iljin K, Bucher E, Sara H, Pisto T, Saarela M, Skotheim RI, Bjorkman M, Mpindi JP, Haapa-Paananen S, Vainio P, Edgren H, Wolf M, Astola J (2008). Systematic bioinformatic analysis of expression levels of 17,330 human genes across 9,783 samples from 175 types of healthy and pathological tissues. Genome Biol.

[81] Allander SV, Illei PB, Chen Y, Antonescu CR, Bittner M, Ladanyi M, Meltzer PS (2002). Expression profiling of synovial sarcoma by cDNA microarrays: association of ERBB2, IGFBP2, and ELF3 with epithelial differentiation. The American journal of pathology.

[82] Feral C, Wu YQ, Pawlak A, Guellaen G (2001). Meiotic human sperm cells express a leucine-rich homologue of Caenorhabditis elegans early embryogenesis gene, Zyg-11. Molecular human reproduction.

[83] Sonneville R, Gonczy P (2004). Zyg-11 and cul-2 regulate progression through meiosis II and polarity establishment in C. elegans. Development.

[84] Liu J, Vasudevan S, Kipreos ET (2004). CUL-2 and ZYG-11 promote meiotic anaphase II and the proper placement of the anterior-posterior axis in C. elegans. Development.

[85] Kemphues KJ, Wolf N, Wood WB, Hirsh D (1986). Two loci required for cytoplasmic organization in early embryos of Caenorhabditis elegans. Developmental biology.

